# Mechanisms Underlying Soybean Response to Phosphorus Deficiency through Integration of Omics Analysis

**DOI:** 10.3390/ijms23094592

**Published:** 2022-04-21

**Authors:** Xiaohui Mo, Guoxuan Liu, Zeyu Zhang, Xing Lu, Cuiyue Liang, Jiang Tian

**Affiliations:** Root Biology Center, State Key Laboratory for Conservation and Utilization of Subtropical Agro-Bioresources, College of Natural Resources and Environment, South China Agricultural University, Guangzhou 510642, China; xhmo@scau.edu.cn (X.M.); liuguoxuanscau@163.com (G.L.); jysx@stu.scau.edu.cn (Z.Z.); xinglu@scau.edu.cn (X.L.)

**Keywords:** soybean, phosphorus, omics, molecular mechanism

## Abstract

Low phosphorus (P) availability limits soybean growth and yield. A set of potential strategies for plant responses to P deficiency have been elucidated in the past decades, especially in model plants such as *Arabidopsis thaliana* and rice (*Oryza sativa*). Recently, substantial efforts focus on the mechanisms underlying P deficiency improvement in legume crops, especially in soybeans (*Glycine max*). This review summarizes recent advances in the morphological, metabolic, and molecular responses of soybean to phosphate (Pi) starvation through the combined analysis of transcriptomics, proteomics, and metabolomics. Furthermore, we highlight the functions of the key factors controlling root growth and P homeostasis, base on which, a P signaling network in soybean was subsequently presumed. This review also discusses current barriers and depicts perspectives in engineering soybean cultivars with high P efficiency.

## 1. Introduction

As an essential nutrient for plants, phosphorus (P) is not only an important component of biomolecules (e.g., nucleic acids, proteins, and phospholipids) but also participates in a set of metabolic processes including photosynthesis and energy conversions [1,2,3]. Plants directly absorb P in the form of phosphate (Pi), including HPO_4_^2−^ and H_2_PO_4_^−^ [4]. However, Pi availability is limited for crop growth in most soils because of its heterogeneous distribution, fixation by microbial activities, and cations (e.g., Al^3+^, Fe^2+^, Ca^2+^) in soils [5,6]. For example, it was estimated that about 40% of cultivated soils faced a low concentration of Pi in the world [7]. Meanwhile, it has been reported that low Pi availability could lead to direct crop yield reductions of 30–40% [8]. In order to meet the demand for P in crops, a large number of Pi fertilizers are applied. It was estimated that approximately 43.4 million tonnes of Pi fertilizers were applied worldwide in 2019, which was 1.3-fold of those in 2000 (http://faostat.fao.org/, accessed on 7 February 2022). However, the utilization efficiency of Pi fertilizers in crops is generally less than 20%, and the rest of them become immobilized P in soils or flow to surface waters (e.g., rivers, ponds, and lakes), resulting in environmental pollution [9]. Excessive application of Pi fertilizers not only results in a series of environmental problems such as eutrophication but also leads to the waste of natural phosphate rock resources [5,6,10]. Plants have evolved a series of morphological, physiological, and molecular strategies to alleviate the constraint of low Pi availability [3,11,12,13]. The adaptive strategies mainly include the induction of high-affinity Pi transporters, reconstruction of root morphology and architecture, accumulation of anthocyanins in shoots/leaves, replacement of phospholipids by glycolipids and sulfolipids in biomembranes, increased root exudates (e.g., organic acids and acid phosphatases), and a symbiotic association with beneficial microbes [11,14]. Among the morphological responses, a remodeling of the root morphology and architecture in response to low-P stress is commonly observed in most plant species, such as increases in lateral root length and root hair density and the formation of shallow root architectures [15,16,17].

The influence of Pi starvation on plant growth is very complicated [3,11]. In this regard, different “omics” approaches are valuable and preponderant in exploring mechanisms underlying the low-Pi stress response in plants due to their rapid progress. Omics mainly include genomics, transcriptomics, proteomics, metabolomics, lipidomics, and ionomics, which provide large-scale insights into Pi starvation responses and are successfully utilized in plants, such as Arabidopsis, tomato (*Solanum lycopersicum*), *Medicago truncatula*, rice, maize (*Zea mays*), and soybean [18,19,20,21,22,23]. The integration of omics data is a promising approach for providing comprehensive and precise changes in plant responses to low-P stress [11].

The soybean (*Glycine max*) is an important source of oil, protein, and micronutrients for humans and animals and is world-widely grown [24]. In 2020, the global harvest area and production of soybeans, grown in more than 80 countries, were approximately 130 million hectares and 360 million tons, respectively (http://faostat.fao.org/, accessed on 7 February 2022). However, most of the soybeans were produced in several countries; despite that, acid soils are widely distributed in the countries such as Brazil and Argentina [25]. In acid soils, low Pi availability is generally considered to be one of the major constraints in soybean growth and yield [26,27]. Thus far, a set of adaptive strategies and their underlying molecular mechanisms have been extensively investigated in soybean through forward and reverse genetic analysis, as well as omics approaches [28,29,30,31,32,33,34]. However, there remains a large gap in the integration of Pi starvation-responsive genes, proteins, and metabolites in order to shed light on complex regulatory mechanisms of soybean adaption to low-P stress. In this review, we have summarized the current advance in the physiological and molecular mechanisms underlying soybean response to P deficiency combining transcriptomics, proteomics, and metabolomics analysis, and highlighted several key factors mediating soybean P efficiency, which could contribute to developing soybean cultivars with enhanced P uptake and utilization efficiency.

## 2. Integration of Omics Analysis Highlighted Complex Responses of Soybean to Low Pi Availability

### 2.1. Identification of Differential Expression Genes in Responses to Pi Starvation

Transcriptomics refers to a comprehensive analysis of the whole set of RNA molecules in cells, tissues, or organisms through high-throughput sequencing techniques, including microarrays and RNA-sequencing (RNA-seq) [35]. With the aid of transcriptomics analysis, differential expression genes (DEGs) have been identified in different soybean organs or tissues, such as the leaves, roots, and nodules [22,36,37,38,39,40,41]. The identification of DEGs in soybean organs or tissues highlighted complex molecular responses of soybean to Pi starvation.

In soybean leaves, three transcriptomics studies were performed [22,37,39]. Among them, comparative transcriptomics analysis on the soybean leaves led to the identification of a total of 1874 DEGs in a P-efficient RIL line (i.e., B20) and 2345 DEGs in a P-inefficient RIL line (i.e., B18) after 7 d of Pi starvation (Table 1) [37]. Furthermore, 138 DEGs exhibited opposite expression patterns between B18 and B20, which were predicted to involve metabolic pathways including transcription, a cellular biosynthetic process, nitrogen metabolic process, lipid metabolic process, and transferase activity [37]. For example, *Glyma.10G193500* was found to be up-regulated only in B18 and was suggested to be associated with the deep-green leaves observed in B18 but not in B20 [37]. However, it was observed that soybean leaves might exhibit different responses to short-term (i.e., 24 h) Pi starvation because most of the DEGs were found to be mainly involved in Pi signaling, nutrient transportation, hormonal and calcium signaling, protein phosphorylation and dephosphorylation, and cell wall modification [39].

In soybean roots, five transcriptomics studies were conducted [22,36,37,40,41]. Three of them compared the DEGs between P-efficient and -inefficient genotypes and, thus, shed light on molecular mechanisms controlling P efficiency in soybean [22,37,41]. For example, *acid phosphatase 2* (*GmACP2*) was up-regulated in the soybean roots of the P-efficient RIL line (i.e., B20) but not in the P-inefficient RIL line (i.e., B18) by transcriptomics analysis [37]. Furthermore, overexpression of *GmACP2* led to the improvement of acid phosphatase activity, suggesting the role of *GmACP2* in mediating the utilization of organic phosphorus [37]. Meanwhile, *ethylene-overproduction protein 1* (*GmETO1*) was up-regulated in the soybean roots of the P-efficient genotype, NN94156, but not in the P-inefficient genotype, Bogao [41]. Furthermore, the overexpression of *GmETO1* enhanced hairy root proliferation and elongation, suggesting that *GmETO1* might play a role in remodeling root morphology [41]. Additionally, the identification of more DEGs through transcriptomics studies could provide important candidate genes for further study. For instance, *Glyma.10G211000*, one of the homologs of *Like-auxin3* (*LAX3*) involved in the lateral root development in Arabidopsis, was significantly up-regulated in Pi starvation, strongly indicating that it would play a role in root architecture remodeling in soybean under low-P stress [40,42].

Transcriptome analysis was also conducted in soybean nodules, and a total of 2055 DEGs were identified, including a set of genes involved in Pi acquisition and mobilization, nitrate/nitrite absorption, and assimilation (Table 1) [38]. It was found that some DEGs exhibited similar responses to Pi starvation in nodules as roots, such as *PT2*/*4*, *SPX1*/*3*/*4*/*7*/*8*, *PAP11*/*20*/*23*, strongly suggesting that similar responses to Pi starvation presented in both soybean roots and nodules [38,40]. However, a group of genes preferred to increase transcripts under P-deficiency only in nodules but not in roots (e.g., *GmPT1*/*5*/*6*/*7*/*13*/*14*, *GmSPX2*/*5*/*9*, and *GmPAP1*/*30*/*31*/*32*/*35*), indicating that a particular Pi signaling pathway might exist in soybean nodules [38,40]. For example, *GmPT5* was found to regulate Pi homeostasis in soybean nodules by mediating Pi translocation from the roots to the nodules [31,43].

### 2.2. Identification of Differentially Accumulated Proteins in Responses to Pi Starvation

Proteomics is the large-scale study of the function of expressed proteins [44]. Proteomics studies have exponentially increased since “proteome” was first termed in 1994, which developed swiftly from the first to the fourth generation, including gel electrophoresis-based, isotopic labelling-based, shotgun- or label-free-based, and targeted proteomics [45]. With the application of proteomics analysis, differentially accumulated proteins (DAPs) have been identified in different soybean genotypes or organs (e.g., leaves, roots, and nodules) [46,47,48,49,50,51,52,53]. The identification of DAPs sheds light on complicated molecular responses of soybean to Pi deprivation at the translational or post-translational levels.

**Figure 1:**
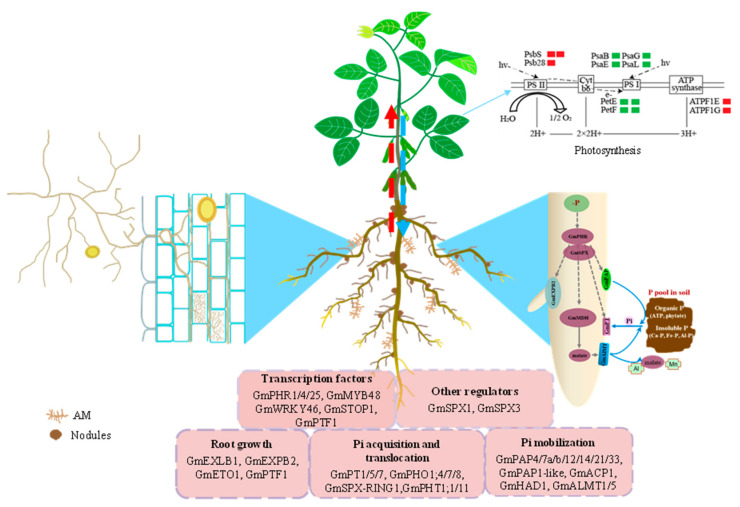
A model of the Pi signaling network in soybean and DAPs involved in photosynthesis. The data on DAPs involved in photosynthesis are from Cheng et al., 2021 [51]. PsbS, photosystem II 22 kDa protein; Psb28, photosystem II 13 kDa protein; PsaB, photosystem I P700 chlorophyll a apoprotein A2; PsaE, photosystem I subunit IV; PsaG, photosystem I subunit V; PsaL, photosystem I subunit XI; PetE, plastocyanin; PetF, ferredoxin; ATPF1E, F-type H^+^-transporting ATPase subunit eps; ATPF1G, F-type H^+^-transporting ATPase subunit gam; red color indicates up-regulated and green color indicates down-regulated. The number of rectangles indicates the number of DAPs. Gm, *Glycine max*.

Three proteomics studies were conducted in soybean shoots or leaves at different P levels [47,49,51]. Among them, two proteomics analyses were conducted through two-dimensional polyacrylamide gel electrophoresis (2-DE), and 37 and 55 DAPs were identified (Table 1) [47,49]. For example, several proteins functioned as key enzymes of the Calvin cycle, such as ribulose-1, 5-bisphosphate carboxylase, rubisco activase, ATP synthase, phosphoenolpyruvate carboxylase, and malate dehydrogenase, which were down-regulated in the plants grown under low-P conditions; this might result in the inhibited photosynthesis capacity and growth of soybean [49]. Furthermore, a label-free quantification (LFQ)-based proteomics was conducted and resulted in the identification of 707 DAPs that were mainly involved in various physiological/biochemical processes, including chlorophyll biosynthetic and carbon metabolic pathways (Table 1) [51]. Among them, 13 DAPs were associated with photosynthesis, and four proteins in photosystem I were significantly down-regulated by Pi deprivation (Figure 1).

In soybean roots, five proteomics studies were conducted [47,48,50,52,53]. Two of them tried to comparatively analyze the DAPs between P-efficient and P-inefficient soybean genotypes by 2-DE and TMT-based proteomics [48,53]. Totally, 660 and 133 DAPs were separately identified in the P-efficient genotype (i.e., Liaodou 13) and P-inefficient genotype (i.e., Tiefeng 3) (Table 1). Among them, the DAPs were predicted to patriciate in carbon metabolism, oxidative phosphorylation, and glycolysis pathways [53]. Furthermore, several DAPs (e.g., I1KW20, I1K3U8, and C6SZ93) were significantly up-regulated only in Liaodou 13 but not in Tiefeng 3, strongly suggesting the DAPs might cause the difference in the P efficiency of two soybean genotypes [53]. Furthermore, a proteomic study with an isobaric tag for relative and absolute quantitation (iTRAQ) was conducted to analyze DAPs in root cell walls [50]. A total of 53 DAPs in the cell walls were identified, including purple acid phosphatase 1-like (GmPAP1-like, Table 1) [50]. Additionally, post-transcriptional modifications (PTMs), including phosphorylation, ubiquitination, and glycosylation, play important roles in regulating protein activity, stability, and localization [54]. Recently, a phosphoproteome was conducted to investigate changes in the phosphoprotein profiles of soybean roots at different P levels, and a total of 427 phosphoproteins were found to be regulated by Pi starvation, with 213 up-regulated and 214 down-regulated proteins (Table 1) [52]. Among them, an auxin efflux transporter, GmPIN2, and a high-affinity nitrate transporter 2.4A, GmNRT2.4A, were identified to be up-regulated by P deficiency, suggesting that auxin and nitrogen acquisition participated in regulating soybean root responses to Pi starvation through protein phosphorylation and dephosphorylation [52].

In soybean nodules, 2-DE-based proteomics was performed to identify DAPs in responses to Pi starvation [46]. Totally, 27 DAPs were identified, including 14 up-regulated and 13 down-regulated proteins, such as two malate dehydrogenases (i.e., GmMDH6 and GmMDH12, Table 1) [46]. Interestingly, GmMDH12 was found to be involved in C and N metabolism in the nodules, and the regulation of the low-P stress inhibited nodule development [30]. Additionally, it was found that 11 of the 14 up-regulated proteins and four of thirteen down-regulated proteins were consistent with the expression pattern of the corresponding genes by qRT-PCR analysis, suggesting that most up-regulated proteins may be mediated at the transcriptional level, while down-regulated proteins may depend on the regulation at the protein level [46]. Therefore, it is necessary to study protein abundance and roles at both the translational and post-translational levels.

### 2.3. Identification of Differentially Accumulation Metabolites in Responses to Pi Starvation

Metabolomics is the large-scale identification and quantitation of metabolites in the cell, tissue, and organism at a specific point in time [55]. Metabolomics has rapidly developed in the past two decades due to an advancement in analytical methods, including gas/liquid chromatography coupled with mass spectrometry (GC-MS/LC-MS) and nuclear magnetic resonance (NMR) [56]. With the aid of metabolomics analysis, differentially accumulated metabolites (DAMs) have been identified in soybean roots and nodules to P deficiency [40,57,58]. The metabolomics provide a precise portrayal of the physiological states of plants and, thus, may reveal the potential mechanisms underlying the phenotypic effects of low-P stress in soybean.

**Table 1:** A list of omics analyses of soybean responses to phosphate starvation.

Omics	Soybean Genotypes	Organ/Tissues	Treatment Time (d)	Methods	Number of DEG/DAP/ DAM (#)	Up-Regulated (#)	Down-Regulated (#)	References
Transcriptomics	Low-P-tolerant accession Chundou	Leaves/Roots	10	Microarray	11/298	11/257	0/41	[22]
	Low-P-sensitive accession Yunhefengwodou				7/3	0/0	7/3	
	Williams 82	Roots	7	RNA-seq	1612	727	885	[36]
	Low-P-tolerant RIL line B20	Leaves/Roots	7	RNA-seq	1874/1286	1284/874	590/412	[37]
	Low-P-sensitive RIL line B18				2345/1150	1113/554	1232/596	
	YC03-3	Nodules	25	RNA-seq	2055	1431	624	[38]
	Williams 82	Leaves	1	RNA-seq	533	303	230	[39]
	YC03-3	Roots	12	RNA-seq	1644	1199	445	[40]
	Low-P-tolerant genotype NN94156	Roots	7	RNA-seq	1280	495	785	[41]
	Low-P-sensitive genotype Bogao				1620	814	806	
Proteomics	HN66	Nodules	25	2-DE MALDI TOF MS	27	14	13	[46]
	BX10	Shoots/Roots	3, 6	2-DE MALDI TOF MS	37/51	23/33	14/18	[47]
	P-efficient genotype EC-232019	Roots	20	2-DE MALDI TOF MS	75	45	30	[48]
	P-inefficient genotype EC-113396				54	34	20	
	Low-P tolerant RIL line B20	Leaves	14	2-DE MALDI-TOF MS	17	7	10	[49]
	YC03-3	Roots	10	iTRAQ LC-MS/MS	71	30	41	[50]
	Williams 82	Leaves	14	LFQ LC-MS/MS	707	267	440	[51]
	YC03-3	Roots	14	iTRAQ LC-MS/MS	427	213	214	[52]
	Low-P-tolerant genotype Liaodou 13	Roots	9	TMT LC-MS/MS	660	656	4	[53]
	Low-P-sensitive genotype Tiefeng 3				133	127	6	
Metabolomics	YC03-3	Roots	12	LC-ESI-MS/MS	155	73	82	[40]
	Jack	Root hairs	7	ESI-MS/MS	16	7	9	[57]
		Stripped root			21	7	14	
	Williams 82	Nodules inoculated with two strains	35	GC-TOF/MS	43/36	25/14	20/25	[58]

d, day; #, the number of DEG/DAP/DAM, and up-regulated or down-regulated DEG/DAP/DAM.

In soybean roots, two transcriptomics studies were conducted, including membrane glycerolipidome and targeted metabolome [40,57]. It is generally considered that phospholipids in bio-membranes could be replaced by sulfo- and galacto-lipids to low-P stress [14,59]. Membrane glycerolipidome revealed comprehensive changes of lipids in the root hairs and in stripped roots without root hairs under P deficiency conditions [57]. The ratio of galactolipids to phospholipids was significantly increased in stripped roots, while the increased ratio in root hairs was less than that in stripped roots [57]. As a central intermediate in glycerolipid metabolism, levels of phosphatidic acid (PA) increased significantly by Pi starvation in root hairs, but not in stripped roots, which might be caused by more sensitivity of membrane glycerolipidomes to Pi starvation in root hairs [57]. Furthermore, 531 metabolites were detected through targeted metabolome analysis, and a total of 155 DAMs were identified in soybean roots, including phosphorylated metabolites (e.g., lipids and nucleic acids), flavonoids, and amino acids (Table 1) [40]. Given that flavonoids play crucial roles in plant growth and resistance to biotic and abiotic stresses, 26 DAMs classified as flavonoids, including fustin, butein, and quercetin, might function in soybean roots to Pi deprivation, which merits further study [40,60].

The metabolomic studies were also conducted in soybean nodules inoculated with different rhizobium strains, such as USDA110 and CB1809, belonging to *Bradyrhizobium diazoefficiens* under both Pi-sufficient and deficient conditions [58]. Soybean nodules infected with CB1809 exhibited a stronger symbiotic N_2_ fixation capacity in response to Pi starvation than USDA110, and a total of 43 and 36 DAMs were identified, respectively, in CB1809- and USDA110- inoculated nodules (Table 1) [58]. The DAMs mainly included the amino acids and urea in USDA110-inoculated nodules and the organic acids, pinitol, 5,6-dihydrouracil, and daidzein, in CB1809- inoculated nodules, suggesting that a symbiotic N_2_ fixation capacity under P deficiency might be associated with the utilization efficiency of the overall carbon (C) budget of symbiosis [58].

## 3. Improving Pi Acquisition through Root Modifications

### 3.1. Optimizing Root Architecture

Roots are the main organ for plants to uptake water and mineral nutrients from soils [61,62]. Therefore, the root system (e.g., tap root, lateral root, and root hair) plays a key role in controlling nutrient acquisition efficiency. Since Pi availability is relatively high in the upper layers of soils, the formation of shallow root architecture is generally considered as a typical strategy for the crop adaptation of P deficiency [17,26,63]. Consistently, it has been found that P-efficient soybean genotypes exhibit a shallow root architecture, while P-inefficient genotypes harbor a deep root architecture [17].

A Pi starvation- responsive cell wall *β-expansin*, *GmEXPB2*, was cloned and functionally characterized to mediate root hair density, lateral root length, and number [64,65,66]. Recently, a basic helix-loop-helix transcription factor, GmPTF1, was found to regulate the expression of *GmEXPB2* and, thus, modify root architecture in responses to P starvation [33]. Therefore, the GmPTF1-GmEXPB2 module was suggested to control the reconstruction of root morphology and architecture to P deficiency in soybean [33,65,66]. Additionally, other regulators have been identified and characterized to regulate the soybean root system, such as the WRKY transcription factor (i.e., GmWRKY46), GmETO1, and 6-phosphogluconate dehydrogenase 1 (i.e., Gm6PGDH1) [41,67,68]. For example, the overexpression of *Gm6PGDH1* resulted in increases in the root length and the number of hairy roots [68]. Meanwhile, 14 quantitative trait loci (QTL) associated with the soybean root system were identified, in which qP10-2 was localized on chromosome 10 and contained a gene encoding *acid phosphatase GmHAD1* [69].

### 3.2. Induction of High-Affinity Pi Transporter

Plant direct Pi absorption from soil solutions is mainly dependent on phosphate transporters (PHTs/PTs), most of which exhibit Pi starvation responses and mediate Pi homeostasis in plants [1,2]. In soybean, two PT genes (i.e., GmPT1 and GmPT2) were firstly reported to function in Pi uptake [70]. Subsequently, 14 PT genes (GmPT1-14) in the soybean genome were identified and characterized [71,72]. Furthermore, overexpressing *GmPT1* led to significant increases in the total dry weight, seed weight, and phosphorus-use efficiency under Pi-deficient conditions, indicating its function in Pi absorption or translocation [73]. Interestingly, several *GmPTs* were found to be expressed in symbiotic soybean with other microorganisms (e.g., rhizobia or arbuscular mycorrhizal fungi), strongly suggesting *GmPTs* might play a vital role in Pi acquisition and transport in symbiosis [43,71,74]. For example, *GmPT5* maintained Pi homeostasis in the nodules by regulating Pi transport from the roots to the nodules and also controlled nodule growth [43]. The suppression of both *GmPT5* and *GmPT7* resulted in almost disappeared nodules and dramatically decreased the N and P content [31].

### 3.3. Activization Pi from Insoluble P Pools

There are two types of P forms in soils, including organic and inorganic P forms. A great part of P is present in immobile forms, such as organic P (e.g., phytate) and insoluble inorganic P (e.g., Ca-P, Fe-P, Al-P), which are not directly acquired by plants [14]. It is usually believed that root exudates, including acid phosphatases (APases), organic acids, and protons (H^+^), are involved in Pi activation from organic P and insoluble inorganic P in the rhizosphere and, thereby, enhance P acquisition efficiency [75,76,77,78,79].

APases are a class of hydrolases that catalyze the hydrolysis of phosphate monoesters or acid anhydrides to release Pi, in which the optimum pH of enzymatic activity is generally lower than 7.0 [80]. As a well-known group of APases, purple acid phosphatases (PAPs) are suggested to be involved in the utilization of extracellular organic P [77,78,79,81]. In soybean, a total of 38 PAP genes were identified, and most of them were significantly up-regulated by Pi starvation [32]. For example, low P up-regulated *GmPAP7a*/*b*, *GmPAP4* (*GmPAP18a*), *GmPAP14* (*GmPAP10a*), and *GmPAP1-like* (*GmPAP1b*), which play important roles in the solubilization and utilization of extracellular ATP, phytate, and dNTPs were enhanced by root-associated APase activities [32,50,82,83]. Furthermore, GmPAP12 (GmPAP12a) and GmPAP21 (GmPAP22b) were suggested to mediate Pi homeostasis in nodules, and GmPAP33 (GmPAP20b) was found to participate in the hydrolysis of phospholipids in senescent arbuscules [84,85,86]. These results suggest the functional diversity and complexity of GmPAP members in dealing with low-P stress. Additionally, GmHAD1 and GmACP1, the members of haloacid dehalogenase (HAD) families, had APase activities and were suggested to control P efficiency in soybean [69,87].

Increased exudation of organic acids or H^+^ plays an important role in the activation of fixed inorganic P in soils [78,79,88,89,90]. In soybean, Pi starvation led to increased synthesis and exudates of organic acids, including malate and citrate [28,78,79,89]. Furthermore, several soybean aluminum-activated malate transporters, GmALMTs (i.e., GmALMT1 and GmALMT5), were found to mediate malate exudation and activate the fixed inorganic P in soils [28,29]. For example, the overexpression of *GmALMT5* significantly increased malate exudation and, thus, improved P absorption in transgenic Arabidopsis supplied with Ca-P as the only P source [29]. However, molecular mechanisms for the regulation of Pi starvation responsive H^+^ exudation remain to be fully revealed in further studies.

### 3.4. Effects of Symbiosis on Soybean P Efficiency

In terrestrial ecosystems, most plants can form symbiotic relationships with arbuscular mycorrhizal fungi (AMF) and, thus, enhance their capability to obtain mineral nutrients, especially P [91,92]. Soybean also form a beneficial symbiotic relationship with AMF, which play an important role in the soybean response to Pi starvation [93]. The biomass and P uptake efficiency were significantly increased in soybean colonized with *Rhizophagus irregularis* under P deficiency [93]. Furthermore, it has been documented that soybean phosphate transporters are involved in Pi transport in the symbiosis of soybean with mycorrhizal fungi, such as GmPT7/10/11 [74]. Meanwhile, GmPAP33, a member of the PAP family, was found to facilitate Pi released from phospholipids in senescent arbuscules and, thus, enhanced Pi uptake and soybean growth [85].

Legumes also establish a symbiotic relationship with rhizobium bacteria and form nodules for biological nitrogen fixation (BNF). It has been well documented that nodule growth and development are suppressed by P deficiency, as reflected by the decreased nodule numbers or size and nitrogenase activity in soybean [34,38,43,46,66]. Several regulators have been identified to control the Pi homeostasis in soybean nodules. For example, two soybean cell wall β-expansin members, GmEXPB2 and GmINS1 (also named GmEXPB6), were found to be involved in nodule BNF capacity under low-P stress, as reflected by an increase in nodule size, N_2_ fixation capacity and, thus, N and P content in transgenic soybean [66,94]. Interestingly, malate dehydrogenase, GmMDH12, and GmPAP12 were suggested to mediate the metabolic process in soybean nodules in response to Pi starvation [30,86]. Recently, a Pi signaling module, GmSPX5–GmNF-YC4–GmASL6 in soybean nodules, has been documented and suggested to control soybean nodule adaptation to low-P stress through mediating the asparagine metabolic pathway [34]. The findings suggest the complicated and specific mechanisms underlying soybean nodule adaptation to Pi starvation, which merits further studies.

## 4. Core Regulators in P Signaling Network

In recent decades, the signaling network underlying plant responses to P deficiency has been well documented in model plants, such as Arabidopsis and rice [95]. Among the participants in the P signaling network, miRNAs, SPX, phosphate starvation response 1 (PHR1), and other related transcription factors are considered central regulators and control the expression of a large portion of the Pi starvation-responsive genes [1,95,96].

In soybean, several regulators have been characterized in the P signaling network. As one of the 35 PHR members, GmPHR25 exhibited high Pi starvation responses in soybean, and the overexpression of *GmPHR25* increased Pi concentration in transgenic soybean hairy roots under Pi-sufficient conditions, strongly suggesting that GmPHR25 plays an important role in controlling Pi homeostasis [97]. Furthermore, GmPHR1 and GmPHR4 regulated the transcripts of *GmPHT1;1* and *GmPHT1;4* by binding with the P1BS cis-element-containing fragments in the promoters of *GmPHT1;1* and *GmPHT1;4*, which mediated the Pi homeostasis in the nodules [98,99]. The other regulator, SPX, was also documented to control soybean response to Pi starvation. In the soybean, a total of nine *GmSPX* members were identified and up-regulated by P deficiency [100,101]. Furthermore, the overexpression of *GmSPX3* increased P concentrations in the roots in P-sufficient conditions probably by up-regulating the expression of several Pi starvation-responsive genes, suggesting that GmSPX3, similar to PvSPX1, is a positive regulator in P homeostasis [100,102,103]. On the contrary, GmSPX1, similar to AtSPX3, is a negative regulator in P homeostasis, and the overexpression of *GmSPX1* led to significant decreases in Pi acquisition and the transcripts of Pi starvation-responsive genes [101,102].

## 5. Conclusions and Perspectives

In past decades, omics analysis elucidated comprehensive responses of soybean to Pi starvation at transcriptional, translational, and post-translational levels. However, the differences in the experimental materials, methods, and duration of low-P stress make it difficult to integrate the information provided by transcriptomics, proteomics, and metabolomics analysis. Therefore, integrated multi-omics (e.g., transcriptomics and proteomics, and transcriptomics and metabolomic) analyses are required to fully understand the metabolic and molecular responses of soybean to Pi starvation. For example, the increased accumulation of arginine and ornithine might be associated with the up-regulated transcription of *aldehyde dehydrogenase 12A1* (*ALDH12A1*) in soybean roots under Pi deprivation [40]. Additionally, the functions of key regulators have been investigated via reverse and forward genetic analysis combined with molecular and biochemical methods. Therefore, the P signaling network in soybean was preliminarily constructed (Figure 1).

Although most of the Pi signaling pathways in soybean were consistent with those in model plants (i.e., Arabidopsis and rice), unique regulatory mechanisms might present in soybean because it can form a symbiosis with both rhizobia and AMF. For example, *GmPT5* and GmPT7 functioned in Pi transport from the roots to the nodules and maintained Pi homeostasis and growth in the nodules [31]. Furthermore, the phosphate starvation responses in the roots were distinct between Arabidopsis and soybean. For example, the growth of tap roots is significantly inhibited by Pi starvation in Arabidopsis mediated by *phosphate deficiency response 2* (*PDR2*) and *low phosphate root* 1/2 (*LPR1*/*2*), which has not been observed in soybean [2,11,40]. Due to the complex genome and inefficient and time-consuming transformation, functional characteristics of the genes involving soybean responses to Pi starvation have lagged behind model plants (e.g., Arabidopsis and rice), and a more intensive investigation is required to illustrate the regulatory network of soybean further. In summary, the above studies have provided comprehensive insights into the current advance of soybean responses to low-P stress and its underlying regulatory mechanisms, which could be applied to the genetic improvement of soybean at both the theory and the practical level. The research focus is shifting from single-gene analysis to system-level analysis combining traditional and emerging genetic engineering techniques for the genetic improvement of soybean varieties with enhanced P efficiency.

## References

[1] Chiou T.J., Lin S.I. (2011). Signaling network in sensing phosphate availability in plants. Annu. Rev. Plant Biol..

[2] Liang C., Wang J., Zhao J., Tian J., Liao H. (2014). Control of phosphate homeostasis through gene regulation in crops. Curr. Opin. Plant Biol..

[3] Ham B.K., Chen J., Yan Y., Lucas W.J. (2018). Insights into plant phosphate sensing and signaling. Curr. Opin. Biotechnol..

[4] Hinsinger P. (2001). Bioavailability of soil inorganic P in the rhizosphere as affected by root-induced chemical changes: A review. Plant Soil.

[5] Beardsley T.M. (2011). Peak Phosphorus. Bioscience.

[6] Veneklaas E.J., Lambers H., Bragg J., Finnegan P.M., Lovelock C.E., Plaxton W.C., Price C., Scheible W., Shane M., White P. (2012). Opportunities for improving phosphorus-use efficiency in crop plants. New Phytol..

[7] Vance C.P. (2001). Update on the state of nitrogen and phosphorus nutrition symbiotic nitrogen fixation and phosphorus acquisition. Plant Physiol..

[8] Vance C.P., Uhde-Stone C., Allan D.L. (2003). Phosphorus acquisition and use: Critical adaptations by plants for securing a nonrenewable resource. New Phytol..

[9] MacDonald G.K., Bennett E.M., Potter P.A., Ramankutty N. (2011). Agronomic phosphorus imbalances across the world’s croplands. Proc. Natl. Acad. Sci. USA.

[10] Ju X., Kou C., Christie P., Dou Z., Zhang F. (2007). Changes in the soil environment from excessive application of fertilizers and manures to two contrasting intensive cropping systems on the North China Plain. Environ. Pollut..

[11] Ajmera I., Hodgman T.C., Lu C. (2019). An integrative systems perspective on plant phosphate research. Genes.

[12] Lopez-Arredondo D.L., Leyva-González M.A., González-Morales S.I., López-Bucio J., Herrera-Estrella L. (2014). Phosphate nutrition: Improving low-phosphate tolerance in crops. Annu. Rev. Plant Biol..

[13] Gutiérrez-Alanís D., Ojeda-Rivera J.O., Yong-Villalobos L., Cardenas-Torres L., Herrera-Estrella L. (2018). Adaptation to phosphate scarcity: Tips from Arabidopsis roots. Trends Plant Sci..

[14] Plaxton W.C., Tran H.T. (2011). Metabolic adaptations of phosphate-starved plants. Plant Physiol..

[15] Li H.B., Xia M., Wu P. (2001). Effect of phosphorus deficiency stress on rice lateral root growth and nutrient absorption. Acta Bot. Sin..

[16] Chevalier F., Pata M., Nacry P., Doumas P., Rossignol M. (2003). Effects of phosphate availability on the root system architecture: Large-scale analysis of the natural variation between Arabidopsis accessions. Plant Cell Envrion..

[17] Zhao J., Fu J., Liao H., He Y., Nian H., Hu Y., Qiu L., Dong Y., Yan X. (2004). Characterization of root architecture in an applied core collection for phosphorus efficiency of soybean germplasm. Chin. Sci. Bull..

[18] Li K.P., Xu C.Z., Li Z.X., Zhang K.W., Yang A.F., Zhang J.R. (2008). Comparative proteome analyses of phosphorus responses in maize (*Zea mays* L.) roots of wild-type and a low-P-tolerant mutant reveal root characteristics associated with phosphorus efficiency. Plant J..

[19] Lan P., Li W., Schmidt W. (2012). Complementary proteome and transcriptome profiling in phosphate-deficient Arabidopsis roots reveals multiple levels of gene regulation. Mol. Cell. Proteom..

[20] Secco D., Jabnoune M., Walker H., Shou H., Wu P., Poirier Y., Whelan J. (2013). Spatio-temporal transcript profiling of rice roots and shoots in response to phosphate starvation and recovery. Plant Cell.

[21] Muneer S., Jeong B.R. (2015). Proteomic analysis provides new insights in phosphorus homeostasis subjected to Pi (inorganic phosphate) starvation in tomato plants (*Solanum lycopersicum* L.). PLoS ONE.

[22] Wang Q., Wang J., Yang Y., Du W., Zhang D., Yu D., Cheng H. (2016). A genome-wide expression profile analysis reveals active genes and pathways coping with phosphate starvation in soybean. BMC Genom..

[23] Liese R., Schulze J., Cabeza R.A. (2017). Nitrate application or P deficiency induce a decline in *Medicago truncatula* N2-fixation by similar changes in the nodule transcriptome. Sci. Rep..

[24] Herridge D.F., Peoples M.B., Boddey R.M. (2008). Global inputs of biological nitrogen fixation in agricultural systems. Plant Soil.

[25] Huerta A.I., Martin A.M. Soybean production costs: An analysis of the United States, Brazil and Argentina. Proceedings of the AAEA Annual Meeting.

[26] Wang X., Yan X., Liao H. (2010). Genetic improvement for phosphorus efficiency in soybean: A radical approach. Ann. Bot..

[27] Zhang Z.H., Palta J.A., Lu P., Ren M.J., Zhu X.T., He J. (2022). Traditional soybean (*Glycine max*) breeding increases seed yield but reduces yield stability under non-phosphorus supply. Funct. Plant Biol..

[28] Liang C.Y., Piñeros M.A., Tian J., Yao Z., Sun L., Liu J., Shaff J., Coluccio A., Kochian L.V., Liao H. (2013). Low pH, aluminum, and phosphorus coordinately regulate malate exudation through GmALMT1 to improve soybean adaptation to acid soils. Plant Physiol..

[29] Peng W., Wu W., Peng J., Li J., Lin Y., Wang Y., Tian J., Sun L., Liang C., Liao H. (2018). Characterization of the soybean GmALMT family genes and the function of GmALMT5 in response to phosphate starvation. J. Integr. Plant Biol..

[30] Zhu S., Chen Z., Xie B., Guo Q., Chen M., Liang C., Bai Z., Wang X., Wang H., Liao H. (2021). A phosphate starvation responsive malate dehydrogenase, GmMDH12 mediates malate synthesis and nodule size in soybean (*Glycine max*). Environ. Exp. Bot..

[31] Chen L., Qin L., Zhou L., Li X., Chen Z., Sun L., Wang W., Lin Z., Zhao J., Yamaji N. (2019). A nodule-localized phosphate transporter GmPT7 plays an important role in enhancing symbiotic N2 fixation and yield in soybean. New Phytol..

[32] Zhu S., Chen M., Liang C., Xue Y., Lin S., Tian J. (2020). Characterization of purple acid phosphatase family and functional analysis of GmPAP7a/7b involved in extracellular ATP utilization in soybean. Front. Plant Sci..

[33] Yang Z., Gao Z., Zhou H., He Y., Liu Y., Lai Y., Zheng J., Li X., Liao H. (2021). GmPTF1 modifies root architecture responses to phosphate starvation primarily through regulating GmEXPB2 expression in soybean. Plant J..

[34] Zhuang Q., Xue Y., Yao Z., Zhu S., Liang C., Liao H., Tian J. (2021). Phosphate starvation responsive GmSPX5 mediates nodule growth through interaction with GmNF-YC4 in soybean (*Glycine max*). Plant J..

[35] McGettigan P.A. (2013). Transcriptomics in the RNA-seq era. Curr. Opin. Chem. Biol..

[36] Zeng H., Wang G., Zhang Y., Hu X., Pi E., Zhu Y., Wang H., Du L. (2016). Genome-wide identification of phosphate-deficiency-responsive genes in soybean roots by high-throughput sequencing. Plant Soil.

[37] Zhang D., Zhang H., Chu S., Li H., Chi Y., Triebwasser-Freese D., Lv H., Yu D. (2017). Integrating QTL mapping and transcriptomics identifies candidate genes underlying QTLs associated with soybean tolerance to low-phosphorus stress. Plant Mol. Biol..

[38] Xue Y., Zhuang Q., Zhu S., Xiao B., Liang C., Liao H., Tian J. (2018). Genome wide transcriptome analysis reveals complex regulatory mechanisms underlying phosphate homeostasis in soybean nodules. Int. J. Mol. Sci..

[39] Zeng H., Zhang X., Zhang X., Pi E., Xiao L., Zhu Y. (2018). Early transcriptomic response to phosphate deprivation in soybean leaves as revealed by RNA-Sequencing. Int. J. Mol. Sci..

[40] Mo X., Zhang M., Liang C., Cai L., Tian J. (2019). Integration of metabolome and transcriptome analyses highlights soybean roots responding to phosphorus deficiency by modulating phosphorylated metabolite processes. Plant Physiol. Biochem..

[41] Zhang H., Yang Y., Sun C., Liu X., Lv L., Hu Z., Yu D., Zhang D. (2020). Up-regulating GmETO1 improves phosphorus uptake and use efficiency by promoting root growth in soybean. Plant Cell Environ..

[42] Lee H.W., Cho C., Kim J. (2015). Lateral organ boundaries domain16 and 18 act downstream of the AUXIN1 and LIKE-AUXIN3 auxin influx carriers to control lateral root development in Arabidopsis. Plant Physiol..

[43] Qin L., Zhao J., Tian J., Chen L., Sun Z., Guo Y., Lu X., Gu M., Xu G., Liao H. (2012). The high-affinity phosphate transporter GmPT5 regulates phosphate transport to nodules and nodulation in soybean. Plant Physiol..

[44] Tyers M., Mann M. (2003). From genomics to proteomics. Nature.

[45] Jorrin-Novo J.V., Komatsu S., Sanchez-Lucas R., Rodríguez de Francisco L.E. (2019). Gel electrophoresis-based plant proteomics: Past, present, and future. happy 10th anniversary journal of proteomics!. J. Proteom..

[46] Chen Z., Cui Q., Liang C., Sun L., Tian J., Liao H. (2011). Identification of differentially expressed proteins in soybean nodules under phosphorus deficiency through proteomic analysis. Proteomics.

[47] Sha A., Li M., Yang P. (2016). Identification of phosphorus deficiency responsive proteins in a high phosphorus acquisition soybean (*Glycine max*) cultivar through proteomic analysis. BBA Proteins Proteom..

[48] Vengavasi K., Pandey R., Abraham G., Yadav R. (2017). Comparative analysis of soybean root proteome reveals molecular basis of differential carboxylate efflux under low phosphorus stress. Genes.

[49] Chu S., Li H., Zhang X., Yu K., Chao M., Han S., Zhang D. (2018). Physiological and proteomics analyses reveal low-phosphorus stress affected the regulation of photosynthesis in soybean. Int. J. Mol. Sci..

[50] Wu W., Lin Y., Liu P., Chen Q., Tian J., Liang C. (2018). Association of extracellular dNTP utilization with a GmPAP1-like protein identified in cell wall proteomic analysis of soybean roots. J. Exp. Bot..

[51] Cheng L., Min W., Li M., Zhou L., Hsu C., Yang X., Jiang X., Ruan Z., Zhong Y., Wang Z. (2021). Quantitative proteomics reveals that GmENO2 proteins are involved in response to phosphate starvation in the leaves of *Glycine max* L.. Int. J. Mol. Sci..

[52] Jiang W., He P., Zhou M., Lu X., Chen K., Liang C., Tian J. (2021). Soybean responds to phosphate starvation through reversible protein phosphorylation. Plant Physiol. Biochem..

[53] Zhao H., Yang A., Kong L., Xie F., Wang H., Ao X. (2021). Proteome characterization of two contrasting soybean genotypes in response to different phosphorus treatments. AoB Plants.

[54] Zhou S., Zhu S., Cui S., Hou H., Wu H., Hao B., Cai L., Xu Z., Liu L., Jiang L. (2021). Transcriptional and post-transcriptional regulation of heading date in rice. New Phytol..

[55] Idle J.R., Gonzalez F.J. (2007). Metabolomics. Cell Metab..

[56] Jorge T.F., Rodrigues J.A., Caldana C., Schmidt R., van Dongen J.T., Thomas-Oates J., António C. (2016). Mass spectrometry-based plant metabolomics: Metabolite responses to abiotic stress. Mass Spectrom. Rev..

[57] Wei F., Fanella B., Guo L., Wang X. (2016). Membrane glycerolipidome of soybean root hairs and its response to nitrogen and phosphate availability. Sci. Rep..

[58] Sulieman S., Kusano M., Ha C.V., Watanabe Y., Abdalla M.A., Abdelrahman M., Kobayashi M., Saito K., Mühling K.H., Tran L.P. (2019). Divergent metabolic adjustments in nodules are indispensable for efficient N2 fixation of soybean under phosphate stress. Plant Sci..

[59] Mehra P., Pandey B.K., Verma L., Giri J. (2019). A novel glycerophosphodiester phosphodiesterase improves phosphate deficiency tolerance in rice. Plant Cell Environ..

[60] Tohge T., de Souza L.P., Fernie A.R. (2017). Current understanding of the pathways of flavonoid biosynthesis in model and crop plants. J. Exp. Bot..

[61] Lambers H., Shane M.W., Cramer M.D., Pearse S.J., Veneklaas E.J. (2006). Root structure and functioning for efficient acquisition of phosphorus: Matching morphological and physiological traits. Ann. Bot..

[62] Liu D. (2021). Root developmental responses to phosphorus nutrition. J. Integr. Plant Biol..

[63] Lynch J.P. (2011). Root phenes for enhanced soil exploration and phosphorus acquisition: Tools for future crops. Plant Physiol..

[64] Guo W., Zhang L., Zhao J., Liao H., Zhuang C., Yan X. (2008). Identification of temporally and spatially phosphate-starvation responsive genes in *Glycine max*. Plant Sci..

[65] Guo W., Zhao J., Li X., Qin L., Yan X., Liao H. (2011). A soybean β-expansin gene *GmEXPB2* intrinsically involved in root system architecture responses to abiotic stresses. Plant J..

[66] Li X., Zhao J., Tan Z., Zeng R., Liao H. (2015). *GmEXPB2*, a cell wall β-expansin gene, affects soybean nodulation through modifying root architecture and promoting nodule formation and development. Plant Physiol..

[67] Li C., Li K., Liu X., Ruan H., Zheng M., Yu Z., Gai J., Yang S. (2021). Transcription factor GmWRKY46 enhanced phosphate starvation tolerance and root development in transgenic plants. Front. Plant Sci..

[68] Li C., Li K., Zheng M., Liu X., Ding X., Gai J., Yang S. (2021). Gm6PGDH1, a cytosolic 6-phosphogluconate dehydrogenase, enhanced tolerance to phosphate starvation by improving root System development and modifying the antioxidant system in soybean. Front. Plant Sci..

[69] Cai Z., Cheng Y., Xian P., Ma Q., Wen K., Xia Q., Zhang G., Nian H. (2018). Acid phosphatase gene GmHAD1 linked to low phosphorus tolerance in soybean, through fine mapping. Theor. Appl. Genet..

[70] Wu Z., Zhao J., Gao R., Hu G., Gai J., Xu G., Xing H. (2011). Molecular cloning, characterization and expression analysis of two members of the Pht1 family of phosphate transporters in *Glycine max*. PLoS ONE.

[71] Qin L., Guo Y., Chen L., Liang R., Gu M., Xu G., Zhao J., Walk T., Liao H. (2012). Functional characterization of 14 Pht1 family genes in yeast and their expressions in response to nutrient starvation in soybean. PLoS ONE.

[72] Fan C., Wang X., Hu R., Wang Y., Xiao C., Jiang Y., Zhang X., Zheng C., Fu Y. (2013). The pattern of Phosphate transporter 1 genes evolutionary divergence in *Glycine max* L.. BMC Plant Biol..

[73] Song H., Yin Z., Chao M., Ning L., Zhang D., Yu D. (2014). Functional properties and expression quantitative trait loci for phosphate transporter GmPT1 in soybean. Plant Cell Environ..

[74] Tamura Y., Kobae Y., Mizuno T., Hata S. (2012). Identification and expression analysis of arbuscular mycorrhiza-inducible phosphate transporter genes of soybean. Biosci. Biotechnol. Biochem..

[75] Tesfaye M., Dufault N.S., Dornbusch M.R., Allan D.L., Vance C.P., Samac D.A. (2003). Influence of enhanced malate dehydrogenase expression by alfalfa on diversity of rhizobacteria and soil nutrient availability. Soil Biol. Biochem..

[76] Liang C., Sun L., Yao Z., Liao H., Tian J. (2012). Comparative analysis of PvPAP gene family and their functions in response to phosphorus deficiency in common bean. PLoS ONE.

[77] Tian J., Liao H. (2015). The role of intracellular and secreted purple acid phosphatases in plant phosphorus scavenging and recycling. Annu. Plant Rev..

[78] Chen Z., Liao H. (2016). Organic acid anions: An effective defensive weapon for plants against aluminum toxicity and phosphorus deficiency in acidic soils. J. Genet. Genom..

[79] Canarini A., Kaiser C., Merchant A., Richter A., Wanek W. (2019). Root exudation of primary metabolites: Mechanisms and their roles in plant responses to environmental stimuli. Front. Plant Sci..

[80] Duff S.M., Sarath G., Plaxton W.C. (1994). The role of acid phosphatases in plant phosphorus metabolism. Physiol. Plant..

[81] Bozzo G.G., Raghothama K.G., Plaxton W.C. (2004). Structural and kinetic properties of a novel purple acid phosphatase from phosphate-starved tomato (*Lycopersicon esculentum*) cell cultures. Biochem. J..

[82] Kong Y., Li X., Ma J., Li W., Yan G., Zhang C. (2014). GmPAP4, a novel purple acid phosphatase gene isolated from soybean (*Glycine max*), enhanced extracellular phytate utilization in *Arabidopsis thaliana*. Plant Cell Rep..

[83] Kong Y., Li X., Wang B., Li W., Du H., Zhang C. (2018). The soybean purple acid phosphatase GmPAP14 predominantly enhances external phytate utilization in plants. Front. Plant Sci..

[84] Li C., Li C., Zhang H., Liao H., Wang X. (2017). The purple acid phosphatase GmPAP21 enhances internal phosphorus utilization and possibly plays a role in symbiosis with rhizobia in soybean. Physiol. Plant.

[85] Li C., Zhou J., Wang X., Liao H. (2019). A purple acid phosphatase, GmPAP33, participates in arbuscule degeneration during arbuscular mycorrhizal symbiosis in soybean. Plant Cell Environ..

[86] Wang Y., Yang Z., Kong Y., Li X., Li W., Du H., Zhang C. (2020). GmPAP12 is required for nodule development and nitrogen fixation under phosphorus starvation in soybean. Front. Plant Sci..

[87] Zhang D., Song H., Cheng H., Hao D., Wang H., Kan G., Jin H., Yu D. (2014). The acid phosphatase-encoding gene GmACP1 contributes to soybean tolerance to low-phosphorus stress. PLoS Genet..

[88] Ryan P., Delhaize E., Jones D. (2001). Function and mechanism of organic anion exudation from plant roots. Annu. Rev. Plant Physiol. Plant Mol. Biol..

[89] Dong D., Peng X., Yan X. (2004). Organic acid exudation induced by phosphorus deficiency and/or aluminium toxicity in two contrasting soybean genotypes. Physiol. Plant..

[90] Haichar F.E.Z., Santaella C., Heulin T., Achouak W. (2014). Root exudates mediated interactions below ground. Soil Biol. Biochem..

[91] Smith S.E., Read D.J. (2008). Mycorrhizal Symbiosis. Q. Rev. Biol..

[92] Genre A., Lanfranco L., Perotto S., Bonfante P. (2020). Unique and common traits in mycorrhizal symbioses. Nat. Rev. Microbiol..

[93] Wang X., Zhao S., Bücking H. (2016). Arbuscular mycorrhizal growth responses are fungal specific but do not differ between soybean genotypes with different phosphate efficiency. Ann. Bot..

[94] Yang Z., Zheng J., Zhou H., Chen S., Gao Z., Yang Y., Li X., Liao H. (2021). The soybean β-expansin gene GmINS1 contributes to nodule development in response to phosphate starvation. Physiol. Plant..

[95] Puga M.I., Rojas-Triana M., de Lorenzo L., Leyva A., Rubio V., Paz-Ares J. (2017). Novel signals in the regulation of Pi starvation responses in plants: Facts and promises. Curr. Opin. Plant Biol..

[96] Wu P., Shou H., Xu G., Lian X. (2013). Improvement of phosphorus efficiency in rice on the basis of understanding phosphate signaling and homeostasis. Curr. Opin. Plant Biol..

[97] Xue Y.B., Xiao B.X., Zhu S.N., Mo X.H., Liang C.Y., Tian J., Liao H. (2017). GmPHR25, a GmPHR member up regulated by phosphate starvation, controls phosphate homeostasis in soybean. J. Exp. Bot..

[98] Li L., Guo N., Wu Z., Zhao J., Sun J., Wang X., Xing H. (2015). P1BS, a conserved motif involved in tolerance to phosphate starvation in soybean. Genet. Mol. Res..

[99] Lu M., Cheng Z., Zhang X., Huang P., Fan C., Yu G., Chen F., Xu K., Chen Q., Miao Y. (2020). Spatial divergence of PHR-PHT1 modules maintains phosphorus homeostasis in soybean nodules. Plant Physiol..

[100] Yao Z., Tian J., Liao H. (2014). Comparative characterization of GmSPX members reveals that GmSPX3 is involved in phosphate homeostasis in soybean. Ann. Bot..

[101] Zhang J., Zhou X., Xu Y., Yao M., Xie F., Gai J., Li Y., Yang S. (2016). Soybean SPX1 is an important component of the response to phosphate deficiency for phosphorus homeostasis. Plant Sci..

[102] Duan K., Yi K., Dang L., Huang H., Wu W., Wu P. (2008). Characterization of a sub family of Arabidopsis genes with the SPX domain reveals their diverse functions in plant tolerance to phosphorus starvation. Plant J..

[103] Yao Z., Liang C., Zhang Q., Chen Z.J., Xiao B., Tian J., Liao H. (2014). SPX1 is an important component in the phosphorus signaling network of common bean regulating root growth and phosphorus homeostasis. J. Exp. Bot..

